# Fighting the Host Reaction to SARS-COv-2 in Critically Ill Patients: The Possible Contribution of Off-Label Drugs

**DOI:** 10.3389/fimmu.2020.01201

**Published:** 2020-05-27

**Authors:** Stefania Scala, Roberto Pacelli

**Affiliations:** ^1^Functional Genomics, Istituto Nazionale per lo Studio e la Cura dei Tumori, “Fondazione G. Pascale” – IRCCS, Naples, Italy; ^2^Department of Advanced Biomedical Sciences, School of Medicine, University Federico II, Naples, Italy

**Keywords:** cytokine, inflammation, offlabel drug use, macrophages, interstitial pneumonia

## Abstract

The severe acute respiratory syndrome coronavirus 2 (SARS-COv-2) is the etiologic agent of the 2019 coronavirus disease (COVID19). The majority of infected people presents flu like symptoms and among them 15–20% develops a severe interstitial pneumonitis (IP) that may eventually evolve in acute respiratory distress syndrome (ARDS). IP is caused by the viral glycoprotein spike (S) binding to the angiotensin converting enzyme 2 (ACE2) expressed on the surface of alveolar pneumocytes. The virus is recognized by the “pattern recognition receptors” (PRR) of the immune cells that release cytokines activating more immune cells that produce a large number of pro-inflammatory cytokines, tissue factors and vasoactive peptides. Affected patients might develop the “cytokine storm syndrome,” a fulminant and fatal hypercytokinaemia with multiorgan failure. In patients infected by SARS-COv-2 increase in T-helper 2 (TH2) cytokines (IL-4 and IL10) are reported in addition to the T-helper 1 (TH1) cytokines (IL1B, IFNγ, IP10, and MCP1) previously detected in other coronavirus infections. Cytokines and other molecules involved in immune response and inflammation are conceivable therapeutic targets for IP and ARDS, improving symptoms and decreasing intensive care unit admissions. To this aim off label drugs may be used taking into consideration the window timing for immunosuppressive drugs in virus infected patients. Some off label therapeutic options and preclinical evidence drugs are herein considered.

In December 2019 the third zoonotic coronavirus outbreak of this century happened in a cluster of Chinese patients most of which customers of a seafood market of Wuhan, a big city in the province of Hubei in China (1). On March 11th WHO officially declared a pandemia status. On May 7th, at the WHO website, 205 Countries, Areas, or Territories of the world with at least one case, a total of about 3,634,000 confirmed cases and more than 251,000 deaths were registered.

The severe acute respiratory syndrome coronavirus 2 (SARS-COv-2) causing the corona virus disease of 2019 (COVID 19) is an about 30 kb single strand RNA beta-coronavirus characterized by a genetic mix originating from two bat and two human coronaviruses (Bat-SARS-like (SL)-ZC45, Bat-SL ZXC21, SARS-CoV, and MERS-CoV) (2). Although SARS-COv-2 is less lethal than SARS- and Middle East respiratory syndrome (MERS)-COv, the viral transmission efficiency is higher, with a supposed basic reproduction number of 2.24–3.58, and a mean incubation time of 6 days (3). In a report on more than 70 thousands patients of the Chinese province of Hubei, the majority of infected symptomatic people presented flu like symptoms (mainly fever and cough), with 15–20% of patients developing a severe interstitial pneumonitis (IP) that could evolve in acute respiratory distress syndrome (ARDS). The case fatality rate in the whole population resulted 2.3% (8 and 15%, for patients older than 70 and 80, respectively). In critical patients 49% of case fatality rate was registered (4). IP is caused by the attack of the virus against the alveolar pneumocytes (APs) through the binding of the viral glycoprotein (spike, S) to the angiotensin converting enzyme 2 (ACE2) expressed on the surface of the APs (5). The virus enters in the host target cells through receptor-mediated endocytosis and quickly replicates; virus release in the extracellular space occurs through either budding or cell death. In the extracellular space the virus is recognized by the PRR of immune cells (6). This process contributes to the virus elimination through an amplification cascade in which the immune cells produce a large number of pro-inflammatory cytokines, tissue factors, and vasoactive peptides. These molecules reach the blood vessel wall causing a burst of nitric oxide, damages to the blood vessels and to the coagulation system (7). Among the most involved cells, macrophages play an important role in acute lung injury, which identify pathogen-associated molecular patterns (PAMP) and trigger innate immunity (8, 9). Macrophages secrete a large number of inflammatory mediators and cytokines, such as tumor necrosis factor-alpha (TNF-α), interleukin-1beta (IL-1β), interleukin-6 (IL-6), inducible nitric oxide synthase (iNOS), and macrophage migration inhibitory factor (MIF). TNF-α can directly damage cells of the pulmonary vascular endothelium, increasing capillary endothelial permeability, causing pulmonary edema, predicted by IL-6 level (10). Progression to Acute Respiratory Distress Syndrome (ARDS) is based on the acute onset of lung inflammation, determined by monocyte/macrophage polarization and function. During active infection, inflammatory monocytes/macrophages (IMMs), and resident macrophages undergo marked phenotypic and functional changes, from M1 proinflammatory (classically activated) to M2 inflammatory-resolving macrophages, with a dynamic continuum through discrete categories. During acute infection, monocytes/macrophages often display a phenotype of classically activated macrophages that mediate antiviral host defenses but also promote lung injury by producing nitric oxide (NO), Reactive Oxygen Species (ROS), IL-1, IL-6, and IL-8 and TNF-α. Simultaneously, some macrophages may become M2 macrophages alternatively activated, exerting anti-inflammatory function and regulating wound healing by producing matrix metalloproteinases (MMPs), growth factors, and anti-inflammatory cytokines, particularly TGF-β. Pro-inflammatory macrophages diminish at the removal of stimulus (11–13).

Evidence of a cytokine storm has been found in severe pneumonitis linked to coronavirus infection (14). Previously, in patients with SARS, IL1B, IL6, IL12, IFNγ, IP10, and MCP1 were found to be increased (15). In patients with MERS, IFNγ, TNFα, IL15, and IL17 were shown to participate in the severity of the pneumonitis (16), and an elevated inflammatory innate immune response has been shown in the lower respiratory tract. Although those cytokines were elevated, down-regulation of genes encoding inflammatory TH1 and TH2 molecules was noted (17). Interestingly, in patients infected by SARS-COv-2, there is an increase in IL1β, IFNγ, IP10, and MCP1, probably leading to activated T-helper-1 (TH1) cell responses, and increased production of T-helper-2 (TH2) immunosuppressive cytokines, such as IL4 and IL10 (18). In particular, a significant increase in IL2, IL7, IL10, G-CSF, IP10, MCP1/CCL2, MIP1A, and TNFα was noted in patients requiring admission to the intensive care unit (ICU) compared to patients with a milder disease. As the infiltrate of monocytes, neutrophils, lymphocytes, and macrophages are the cellular actors of the inflammatory response (14), chemokine ligands and receptors play an important role in driving immune cell migration and homing (19). These cytokines may explain the observation of reduced levels of circulating lymphocytes. Peripheral blood examinations on admission in the majority of patients with COVID-19 displayed lymphopenia, elevated infection-related biomarkers (i.e., procalcitonin, erythrocyte sedimentation rate, serum ferritin, and C-reactive protein) (20) and several elevated inflammatory cytokines (i.e., tumor necrosis factor (TNF)-α, interleukin (IL)-2R and IL-6). Patients with more severe cases had higher leukocyte and neutrophil count, lower lymphocyte count and higher neutrophil-to-lymphocyte ratio (NLR) (21). Lymphocyte subsets showed that the total number of B cells, T cells and NK cells was significantly decreased in patients with COVID-19, more significantly so in severe cases. In particular, T cells (T helper, T suppressor, and Tregs cells) were mostly affected by SARS-CoV-2. In addition, recent evidence in SARS-CoV infection suggests that seroconversion may also play a role in lung injury. A detrimental role of early appearance of anti–spike (S)-IgG was demonstrated during SARS-CoV infection in a macaque model (22). Despite markedly reducing virus titers, anti–S-IgG caused lung injury during the early stages of infection, impairing the wound-healing macrophage response and TGF-β production, while promoting pro inflammatory cytokine IL-8, MCP1 production, and inflammatory macrophage accumulation (22). Interestingly, in SARS patients who died in Hong Kong during the 2002 outbreak, the anti-spike (S) glycoprotein neutralizing antibodies appeared significantly before and reached a higher titer than in patients surviving (23). Consistently, preexisting serum antibodies, derived by exposition to influenza seasonal strains, may recognize but fail to neutralize, the new pandemic strain and were found to associate with worse clinical severity during the 2009 influenza pandemic (24, 25).

The inflammatory status together with pulmonary edema and respiratory failure define the clinical picture of the ARDS associated with COVID-19 (26). The most compelling emergency that the health system faces in this epidemic is the shortage of critical care units. The saturation of intensive care units (ICU) precludes the rescue of patients who might be saved, increases COVID-19 lethality rate and worsens the prognosis for other pathological conditions requiring ICU admission. The severe IP or ARDS of the COVID-19 requires ventilator support and can kill infected people averaging in 2 weeks from the appearance of the first symptoms (27, 28). Therapy in use for HIV and other viral disease have been empirically administered without much benefit (29), while promising experimental antiviral drugs such as remdesivir and chloroquine, an old antimalarial drug with *in vitro* activity on the viral infection, are currently in clinical trials (30, 31). In the absence of specific validated approaches, and waiting for a vaccine, a clinical empirical rational management is needed. Another reasonable approach would be drugs targeting the host immune-inflammatory reaction. Methylprednisolone, although somewhat controversial, seems to be overall useful in these patients (32), while dexamethasone has been shown to be useful in patients with ARDS of different etiologies (32, 33).

Cytokines and the other molecules involved in the immune response regulation and inflammation are conceivable targets to improve IP and ARDS lung injury. To this aim off label drugs may be used considering the timing for immunosuppressive drugs in virus infected patients. Unfortunately, the time window is not univocally defined and data may derive from clinical studies.

Several therapeutic options that could be rapidly translated to clinical trials are available. Some of them are listed below.

## Tocilizumab

Tocilizumab is an anti-IL6 receptor antibody (RoActemra, Roche) approved to treat moderate to severe rheumatoid arthritis (RA). Tocilizumab has been used to counteract the side effects of immune checkpoint inhibitors and CAR-T therapy in cancer bearing patients (34) and, recently, to antagonize the host reaction in patients affected by ARDS linked to COVID 19 (35). At today COVID-19 national management guidelines of Chinese health authorities include the use of Tocilizumab for severe pneumonia. A preliminary report on 21 critical cases of COVID-19 suggests efficacy of the treatment with faster recovery and lower risk of death for treated patients, while no toxicity was associated with the reported administration schedule (one or maximum two doses) (36). Timing of administration seems to be crucial as tocilizumab may be more efficient if administered earlier than actual use (37).

## Anakinra

Anakinra is an interleukin-1 receptor antagonist (IL-1RA) previously evaluated in clinical trials for RA patients. IL-1beta/IL-1alpha are two stimulating cytokines of monocyte-macrophage cells acting upstream of the inflammatory signaling pathway induced by inflammasome, thus anakinra should block the cytokine storm. In a small open-label study, anakinra has been tested as agent preventive of mechanic ventilation in 9 patients hospitalized for moderate-severe COVID-19. Amelioration of oxygen flow and blood inflammation markers was described without significant toxicity (38). In clinically moderate and severe COVID-19 patients preliminary evidence reported high levels of three cytokines, CXCL10, CCL7 and IL-1, rather than IL-6, (39). In chronic use Anakinra could determine reaction at the site of injection and infection as the main side effects (40).

## Emapalumab

Emapalumab is a fully human IgG1 monoclonal antibody that binds free and receptor-bound interferon-γ. Emapalumab is approved by the US FDA for the treatment of haemophagocytic (HLH) (41) a rare disorder characterized by pathologic immune activation and hyperinflammation that eventually damage multiple organs. A prospective study has shown a good safety profile of emapalumab in pediatric and adolescent patients affected by HLH, with the infection susceptibility being the major threat (42). Blocking IFN γ activity could counteract the host immune hyper-reaction to SARS-COv-2.

## Mycophenolate

Mycophenolic acid has been used as immunosuppressant agent in pemphigus as a corticosteroid-sparing agent and in kidney transplant patients to avoid rejection. It inhibits inositol monophosphate dehydrogenase, that causes depletion of guanosine and deoxyguanosine nucleotide pools impairing the activity of B and T lymphocytes. The drug has also been demonstrated to inhibit mRNA expression of pro-inflammatory cytokines TNF-α, IL-6, and IL-1β4 (43). Mycophenolic acid has been shown to have activity *in vitro* against zika virus replication (44) and coronavirus through a non-competitive inhibition of MERS-CoV papain-like protease (45). Urinary infections, diarrhea, and leukopenia are the side effects more often described (46).

## Infliximab and Etanercept

Anti-TNFα agents used in autoimmune diseases, such as RA and ulcerative colitis, in principle, may have a role in treating severe respiratory syndrome of COVID-19. Infliximab is a monoclonal antibody targeting TNF alpha while Etanercept is a receptor fusion protein (Human IgG1-Fc plus soluble p75 TNF alpha extracellular domain). TNF-α is a proinflammatory cytokine produced by macrophages, lymphoid cells, endothelial cells, cardiac myocytes, adipose tissue, and brain cells such as microglia and astrocytes. Its receptors are widely expressed and TNF-α plays a key role in immunological defense processes such as inducing fever, inhibiting viral replication during infections, and leading to a permanent growth arrest in cancer (47, 48). Toxicity profile includes augmented risk of infections (49).

## Proteasome Inhibitors

Proteasomal system regulates different cell functions, among which nuclear factor kB (NF-kB) key transcription factor for innate and adaptive immunity (50). Bortezomib inhibits proteasome and it is used in the treatment of myeloma and mantle cell lymphoma. It has been shown to have antiviral activity against herpes virus, targeting viral entry, replication, and assembly (51). Another proteasome inhibitor, VR23, possess powerful anti- inflammatory activity reducing IL-6 in synovial cells from RA patients, and improving LPS-induced acute lung injury by decreasing neutrophil migration, TNF-α secretion, and tissue inflammation in a mice model (52). The dose-limiting toxicity of proteasome inhibitors is the peripheral neuropathy (53) a clinically relevant complication, which negatively impacts the quality of life of multiple myeloma survivors (54).

## PARP-Inhibitors

Pandemic viruses decrease type I interferon (IFN) abundance (24). In humans 17 different types of poly-adenosine 5′-diphosphate (ADP)-ribose polymerase (PARP) are recognized. PARPs transfer ADP ribose from nicotinamide adenine dinucleotide (NAD+) to targeted proteins achieving a post translational modification called ADP-ribosylation, generally in response to stress conditions such as DNA damage, heat shock and viral attack (55). PARP11 is an ADP ribosyl-transferase that inhibits interferon type I (IFN-I) antiviral activity. IFN-I is a key component of the immune response against viral pathogens that induces the expression of several genes (Interferon Stimulated Genes –ISGs) with diverse antiviral properties (56). PARP11 inhibitor, rucaparib has been shown to restore the activity of IFN-I against different viruses in a murine model (57). There is evidence that ZIKV infection triggers type I IFN production by host cells, ZIKV is sensitive to the antiviral activity of IFN and IFN I seems crucial also in SARS-COv-2 infection (58, 59). PARP inhibitors are used in subgroup of patients with breast or ovarian cancer. Toxicity is mainly hematological (60).

## PPARγ Agonists

Peroxisome proliferator-activated receptor gamma (PPARγ- agonists, rosiglitazone and pioglitazone, are drugs in clinical use for diabetes (61). Insulin resistance amplifies inflammation, associated with an increase in C-reactive protein, IL-6, and TNF-α (62) and produces a pro-coagulant state with increased fibrinogen and plasminogen activator inhibitor, (PAI-1) (63). Pioglitazone, in clinical studies on diabetic patients, was able to reduce the plasma level of different inflammatory factors among which CPR, IL-1, IL-6, and TNF-α (64). Thus, it is of great interest that pioglitazone can produce an anti-inflammatory effect also on lung inflammation and fibrosis (65). Considered the excellent tolerability, PPARγ agonists may be tested for amelioration of virus induced lung injuries.

## Plerixafor

Plerixafor is a CXCR4 antagonist used for stem cell mobilization in patients undergoing autologous stem cell transplantation. CXCR4-mediated inflammatory responses is based on the efficient chemotaxis function of inflammatory cells, such as neutrophils, lymphocytes, and monocytes (66).

In murine models of acute lung insufficiency CXCR4 expression was significantly increased in macrophages sorted from bronchoalveolar lavage fluid and receptor downregulation reduced IL-6 and TNF-α. Administration of AMD3100 significantly attenuated the influx of inflammatory cells to the airway and reduced the levels of IL-4, IL-5, and IL-13 in an murine asthmatic model either in the lavage fluid and lung homogenate through attenuation of the Th17 (67), cell population. No adverse events have been described for a single injection of plerixafor (68).

## Sphingosine-1-Phosphate (S1p) Receptors Agonists

Fingolimod, a sphingosine-1-phosphate (S1p) receptor agonist is approved for the treatment of multiple sclerosis (MS). S1p is mainly expressed in vascular endothelial cells and lymphocytes in lung tissue. S1p1 agonists (CYM-5442 and RP-002) have been reported to protect mice from death caused by severe influenza infection, attenuating cytokine production and inhibiting infiltration of innate immune cells. In a mouse model of 2009 H1N1 pandemic influenza, the S1p1 receptor agonist significantly inhibited synthesis of IL-1α, IL-1β, IL-6, IL-10, MCP-1, TNF-α, and GM-CSF, and reduced deaths from lethal infections by more than 80%. In addition the combination of oseltamivir can reduce mouse mortality by 96% (69). Recently a Multiple sclerosis (MS) patient in treatment with fingolimod that was diagnosed with COVID-19 reported a favorable outcome (70). As reported, the toxicity profile even for long term use, is reassuring (71).

In conclusion, while specific antiviral therapies are in rapid development (remdesivir, chloroquine, vaccine), controlling the powerful inflammatory response causing severe IP or ARDS is a reasonable approach. Agents that are available now to improve the lung injuries due to the host reactions and reduce the lethality of the disease are badly needed, and some are already in clinical studies. Drugs targeting multiple cyto/chemokines involved in SARS-COv-2 IP are available for trial or for off-label use, but close attention is needed to the schedule of administration, considered the immunosuppressive action of these drugs. To this aim rapid identification of prognostic factors in the peripheral immune profile may support therapeutic approach. Careful clinical studies are warranted.

## Author Contributions

SS and RP both equally contributed in designing and realizing the manuscript.

## Conflict of Interest

The authors declare that the research was conducted in the absence of any commercial or financial relationships that could be construed as a potential conflict of interest.
